# Clinical Utility of Drug-Eluting Beads Bronchial Arterial Chemoembolization in Advanced Refractory Non-Small Cell Lung Cancer

**DOI:** 10.3390/curroncol33100600

**Published:** 2026-10-05

**Authors:** Yawei Li, Junqing Xi, Yuxi Liu, Xiao Li, Xiaowu Zhang

**Affiliations:** Department of Interventional Therapy, National Cancer Center/National Clinical Research Center for Cancer/Cancer Hospital, Chinese Academy of Medical Sciences and Peking Union Medical College, Beijing 100021, China; mz2v@163.com (Y.L.); xijunqing301@hotmail.com (J.X.);

**Keywords:** drug-eluting beads, bronchial arterial chemoembolization, advanced non-small cell lung cancer, anlotinib, survival

## Abstract

**Objective:** This study mainly focused on evaluating the efficacy and safety of drug-eluting beads bronchial arterial chemoembolization (DEB-BACE) in treating advanced refractory non-small cell lung cancer (NSCLC) patients, with an exploratory investigation of whether combining DEB-BACE with anlotinib could bring additional benefits and identification of key prognostic factors. **Methods:** A retrospective analysis was conducted on 104 eligible patients, including 64 who received DEB-BACE monotherapy and 40 who received DEB-BACE combined with anlotinib. Kaplan–Meier method, log-rank test, and Cox proportional hazard model were used for survival and prognostic analyses. Primary endpoints were progression-free survival (PFS) and overall survival (OS); secondary endpoints included objective response rate (ORR), disease control rate (DCR), and adverse events (AEs). **Results:** The entire cohort had a median PFS of 13.9 months and median OS of 17.0 months, suggesting a potential survival benefit of DEB-BACE in advanced NSCLC. As an exploratory analysis, the combination group showed numerical advantages in PFS (13.9 vs. 10.6 months), OS (20.3 vs. 14.0 months), and ORR, without significant differences (all *p* > 0.05). ECOG 2 was an independent poor prognostic factor for PFS (*HR* = 2.602, *p* = 0.020) and tended to affect OS (*HR* = 2.259, *p* = 0.050). No grade 4 AEs or treatment-related deaths occurred; the 5.8% grade 3 AEs were all manageable. **Conclusions:** DEB-BACE may be effective and safe as a treatment option for advanced NSCLC patients, although prospective controlled studies are needed to confirm these findings. The combination of DEB-BACE with anlotinib, as an exploratory strategy, shows numerical benefits but no significant superiority.

## Simple Summary

Lung cancer is the world’s leading cause of cancer death. For patients with advanced non-small cell lung cancer who no longer respond to standard treatments, effective options are very limited. This study evaluated a minimally invasive procedure called drug-eluting beads bronchial arterial chemoembolization (DEB-BACE), which delivers chemotherapy directly to lung tumors while blocking their blood supply. We reviewed data from 104 patients who received this treatment, some of whom also took an oral targeted therapy called anlotinib. The results suggested that DEB-BACE might be associated with prolonged survival and generally manageable side effects. While adding anlotinib showed some numerical improvements in outcomes, the differences were not statistically significant. Patients with poorer physical function had worse prognoses. These findings suggest that DEB-BACE may be a promising treatment option for this hard-to-treat patient population; however, because this was a retrospective study without a control group, the results should be interpreted with caution.

## 1. Background

According to the latest 2024 report, lung cancer is recognized as the most common type of cancer globally and continues to be the leading cause of cancer-related deaths. In 2022, an estimated 1.8 million people died from lung cancer, which accounted for 18.7% of all global cancer deaths [1]. Non-small cell lung cancer (NSCLC) represents approximately 80–85% of all lung cancer cases in China [2,3]. A vast majority of NSCLC patients are diagnosed at an advanced stage, often accompanied by local invasion or distant metastasis, thus missing the chance for curative surgery [4,5]. Systemic chemotherapy, targeted therapy, and immunotherapy are the mainstay treatment modalities for advanced NSCLC [6]. Nevertheless, drug resistance and intolerance are frequently encountered challenges in clinical practice [7,8,9]. For patients with advanced NSCLC who are either unresponsive to standard treatments or ineligible for them, the available treatment options are rather limited.

Drug-eluting beads bronchial arterial chemoembolization (DEB-BACE) represents a novel interventional treatment technique. It can embolize the tumor-feeding arteries while continuously releasing chemotherapeutic drugs, which not only improves the local drug concentration but also reduces systemic toxicity [10]. Several preliminary studies have demonstrated that DEB-BACE provides clinically meaningful antitumor activity in advanced NSCLC, with reported median progression-free survival (PFS) ranging from 3.0 to 6.3 months and median overall survival (OS) from 8.0 to 15.6 months [11,12,13,14]. However, the long-term efficacy of using DEB-BACE alone remains controversial. Therefore, combination strategies incorporating systemic therapy may further improve therapeutic outcomes.

Anlotinib is an oral small-molecule multi-target tyrosine kinase inhibitor (TKI) that acts on vascular endothelial growth factor receptor (VEGFR), platelet-derived growth factor receptor (PDGFR), and other kinases. This compound exhibits both anti-angiogenic and anti-tumor proliferation properties [15,16]. Having been approved for the third-line treatment of advanced NSCLC, anlotinib has demonstrated favorable efficacy and safety profiles [17,18].

Consequently, in this study, we retrospectively analyzed the efficacy and safety of DEB-BACE in patients with NSCLC who failed or progressed after standard treatment. Moreover, we further explored the value of combining DEB-BACE with anlotinib, aiming to provide evidence to support clinical decision-making for this high-risk patient population.

## 2. Methods

### 2.1. Study Designs and Participants

**Figure 1:**
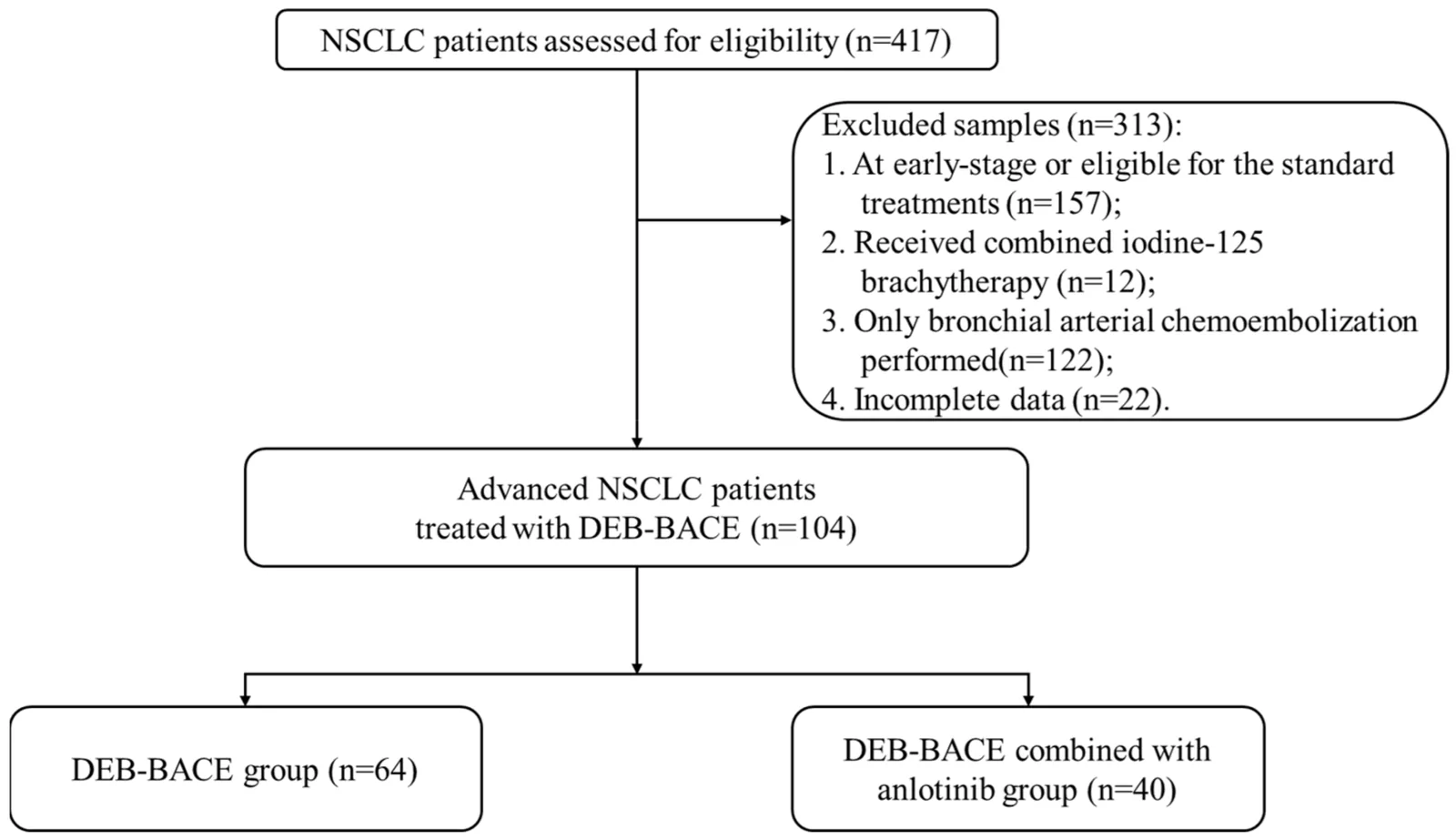
Flowchart of the patient selection. NSCLC, non-small cell lung cancer; DEB-BACE, drug-eluting beads bronchial arterial chemoembolization.

This retrospective study enrolled 417 consecutive NSCLC patients who underwent interventional treatment at our department between January 2018 and December 2025. Among them, 104 patients met the inclusion criteria and received either DEB-BACE plus anlotinib or DEB-BACE alone. Inclusion criteria were as follows: (1) confirmed advanced (stage III–IV) NSCLC, refractory to or ineligible for standard treatment; (2) Eastern Cooperative Oncology Group (ECOG) performance status score of 0–2; (3) presence of at least one measurable intrathoracic lesion; (4) initiation of anlotinib within 1 month after DEB-BACE. Exclusion criteria included: (1) history of other malignant tumors; (2) inadequate medical data (including missing follow-up information); (3) severe dysfunction of the liver, kidney, heart, or hematopoietic system contraindicating interventional or targeted therapy, as shown in Figure 1. Additionally, comprehensive documentation of pathological subtype, TNM stage, tumor size, quantity, location, recurrence/progression status, treatment details, and adverse events was required. Treatment selection (monotherapy vs. combination) was determined by shared decision-making between clinicians and patients, taking into account disease burden, performance status, financial constraints, and patient preference after full discussion of potential risks and benefits of adding anlotinib. The study protocol was approved by the Institutional Ethics Committee of the National Cancer Center, Chinese Academy of Medical Sciences; a waiver of written informed consent was granted due to the retrospective nature of the study (NO. 26/113-0439).

### 2.2. DEB-BACE Treatment Protocol

All enrolled patients underwent DEB-BACE under DSA guidance (Philips Allura Xper FD20, Best, The Netherlands) by interventional radiologists with at least 5 years of experience in thoracic interventional therapy. Under local anesthesia with 2% lidocaine, the right femoral artery was punctured using the Seldinger technique, followed by insertion of a 5-French pigtail catheter into the thoracic aorta for initial angiography to identify the origin, course, and anatomical variations in the feeding arteries, as well as to exclude collateral blood supply to normal tissues (e.g., spinal artery). Under DSA guidance, a 5-French cobra catheter and a microcatheter were used for super-selective catheterization of the target feeding artery to reduce ectopic embolization risk and ensure accurate drug delivery. DC Bead™ (Boston Scientific Corporation, Marlborough, MA, USA, 300–500 μm particle size) was used as drug-eluting beads, selected based on arterial characteristics and tumor vascularity (consistent with 300–500 μm CalliSpheres^®^ beads (Suzhou Hengrui Callisyn Biomedical Co., Ltd., Suzhou, China) per package specifications). Chemotherapeutic agents (gemcitabine 100 mg) were loaded onto the beads according to patients’ treatment history and pathological conditions, and injection was stopped when tumor staining disappeared or contrast agent stagnation was observed.

### 2.3. Administration of Anlotinib

In the combined-treatment group, all patients received oral anlotinib (Chia Tai Tianqing Pharmaceutical Group Co., Ltd., Lianyungang, China; 12 mg per capsule). Anlotinib administration was initiated within 1 week after the initial DEB-BACE therapy, with a standard dose of 12 mg taken orally once daily with warm water, preferably at a consistent morning time. The treatment followed a “2-week-on, 1-week-off” cycle: patients took the medication continuously for 14 days, followed by a 7-day break, which constituted one complete treatment cycle.

### 2.4. Outcomes and Follow-Up

The data cutoff date for survival analysis was 31 January 2026. Tumor response was evaluated by contrast-enhanced chest CT performed one month after treatment and then monthly thereafter, according to RECIST 1.1 criteria; PET-CT was optionally used for suspected metastatic lesions. All imaging assessments were independently reviewed by two radiologists. Adverse events were monitored through routine physical examinations and laboratory tests, and were graded per NCI CTCAE v5.0. Survival status and causes of death were confirmed via medical records or family reports. Tumor responses were categorized based on changes in target lesion size, status of non-target lesions, and the appearance of new lesions, into five categories: complete response (CR), partial response (PR), stable disease (SD), progressive disease (PD), and not evaluable (NE). The primary endpoints were progression-free survival (PFS), defined as the time from the first DEB-BACE procedure to disease progression, death, or last follow-up, whichever occurred first; and overall survival (OS), defined as the time from the first DEB-BACE procedure to death or last follow-up, whichever occurred first. Secondary endpoints included objective response rate (ORR; proportion of patients achieving CR or PR), disease control rate (DCR; proportion of patients achieving CR, PR, or SD), treatment discontinuation due to intolerable adverse events, and death. We acknowledge that PFS and OS may be influenced by systemic disease control; however, these are universally accepted regulatory endpoints for pivotal trials. Given the retrospective design, we have also reported local tumor response per RECIST 1.1 to reflect intrathoracic disease control.

### 2.5. Statistical Analyses

Statistical analyses were carried out using R (version 4.4.1). OS was calculated from the date of the first DEB-BACE procedure to the date of death from any cause or the date of last follow-up, whichever occurred first. PFS was calculated from the date of the first DEB-BACE procedure to the date of documented disease progression, death from any cause, or last follow-up, whichever occurred first. For continuous variables, those with a normal distribution were presented as the mean ± standard deviation, and those with a skewed distribution were shown as the median (interquartile range, IQR). Categorical variables were expressed as *n* (%). Survival analysis was conducted via the Kaplan–Meier method, and comparisons were made using the log-rank test. Prognostic factors were analyzed with Cox proportional hazard models. By integrating the results of univariable analyses and clinical knowledge, variables with *p* < 0.05 were included in subsequent multivariable Cox analyses. A *p*-value of less than 0.05 was regarded as statistically significant.

## 3. Results

### 3.1. Patient Characteristics

A total of 104 eligible patients with NSCLC were enrolled, including 64 in the DEB-BACE monotherapy group and 40 in the DEB-BACE plus anlotinib combination group, with baseline characteristics summarized in Table 1. The combination group had a higher proportion of patients with ECOG 2 (40.0% vs. 18.8%) and a lower proportion with ECOG 1 (25.0% vs. 57.8%), a significantly higher percentage of stage IV disease (70.0% vs. 48.4%), and a greater proportion of patients who had received third- or fourth-line treatment (87.5% vs. 68.8%). The median age of all patients was 67.5 years (IQR 61.0–74.5), with no significant difference (*p* = 0.810). No significant differences were observed between groups in gender, alcohol consumption, body mass index, hypertension, diabetes, cardio-cerebrovascular diseases, pathological subtype, preoperative symptoms, tumor number, tumor distribution, maximum intrapulmonary lesion diameter, intervention number, microsphere diameter, malignant pleural effusion, laboratory parameters (white blood cell count, platelet count, prothrombin time, hemoglobin, CYFRA21-1, NSE, CEA), or extrapulmonary metastases (lymph node, liver, brain metastases; all *p* > 0.05).

### 3.2. Survival Outcomes

**Figure 2:**
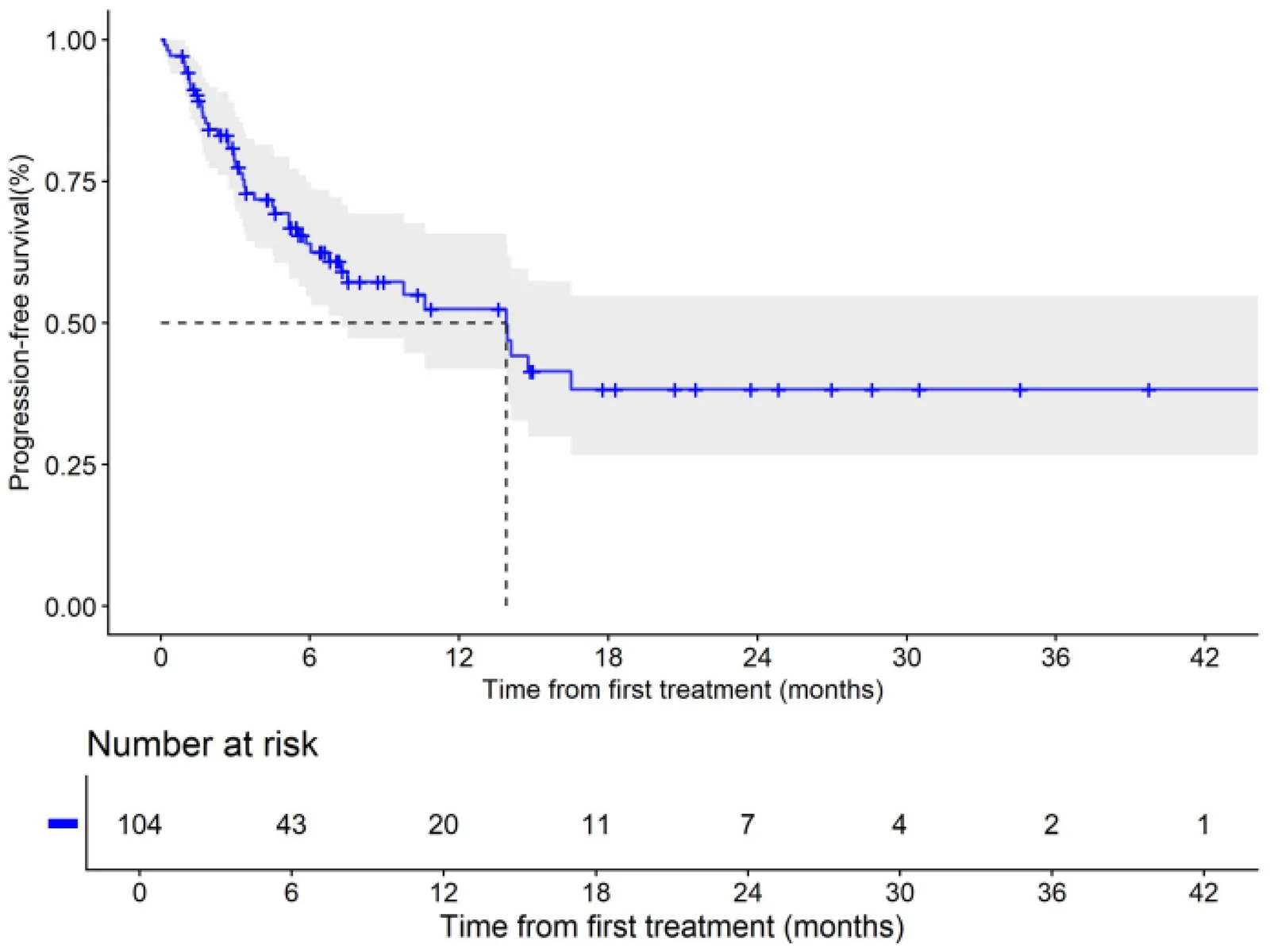
Kaplan–Meier curves for PFS in advanced NSCLC patients treated with DEB-BACE. NSCLC, non-small cell lung cancer; DEB-BACE, drug-eluting beads bronchial arterial chemoembolization; PFS, progression-free survival.

During follow-up, disease progression occurred in 44 of 104 patients (39.4%), 36 patients died (34.6%), and 3 patients were lost to follow-up. The median PFS for all patients was 13.9 months (95% CI: 7.27, NA), and the median OS was 17.0 months (95% CI: 14.0, NA), as shown in Figure 2. The 6-month, 12-month, and 24-month OS rates were 83.9% (95% CI: 76.7–91.8%), 66.5% (95% CI: 55.6–79.6%), and 32.3% (95% CI: 20.2–51.7%), respectively. The median PFS was 13.9 months (95% CI: 5.17, NA) in the DEB-BACE plus anlotinib group and 10.6 months (95% CI: 7.27, NA) in the DEB-BACE monotherapy group, with no significant difference (log-rank *p* = 0.669). The median OS was longer in the combination group (20.3 months; 95% CI: 14.9, NA) than in the monotherapy group (14.0 months; 95% CI: 10.6, NA), although this difference did not reach statistical significance (log-rank *p* = 0.263).

### 3.3. Multivariable Survival Analysis

**Figure 3:**
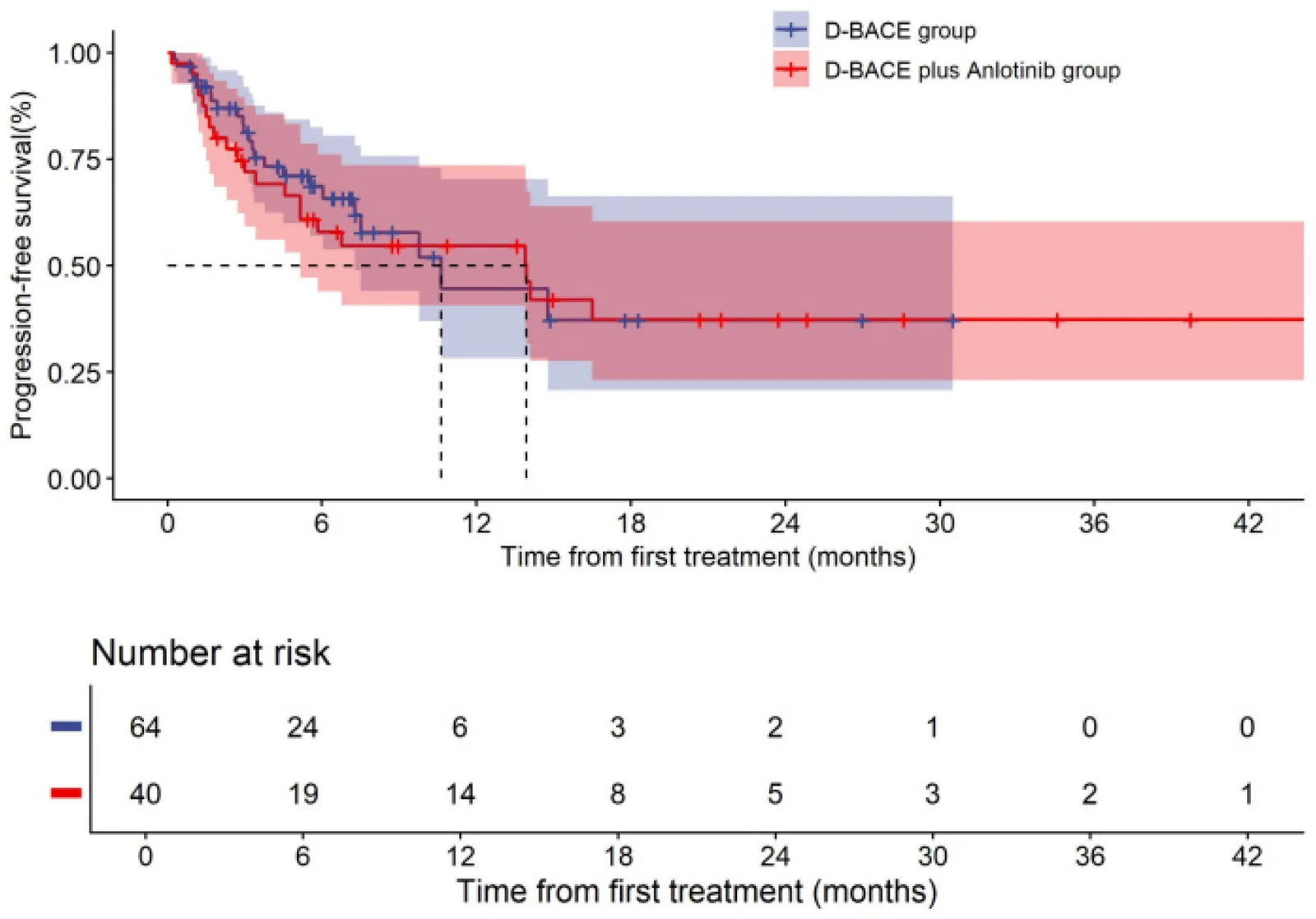
Kaplan–Meier plots of PFS in the DEB-BACE monotherapy group and the combination group. The median PFS was 10.6 months in the DEB-BACE monotherapy group and 13.9 months in the combination group. DEB-BACE, drug-eluting beads bronchial arterial chemoembolization; PFS, progression-free survival.

Multivariable Cox regression analyses revealed independent prognostic factors for PFS. Compared with ECOG 0, ECOG 2 was an independent poor prognostic factor for PFS (*HR* = 2.602, 95% CI: 1.164–5.817, *p* = 0.020), whereas ECOG 1 showed no significant association (*HR* = 1.404, 95% CI: 0.630–3.129, *p* = 0.407). T4 stage tended to be associated with inferior PFS relative to T3 (*HR* = 1.820, 95% CI: 0.902–3.671, *p* = 0.094). Neither DEB-BACE plus anlotinib versus DEB-BACE alone (*HR* = 0.921, 95% CI: 0.474–1.789, *p* = 0.808) nor third- or fourth-line versus second-line treatment (*HR* = 1.022, 95% CI: 0.449–2.327, *p* = 0.958) was significantly associated with PFS, as shown in Table 2, Figure 2 and Figure 3.

**Figure 4:**
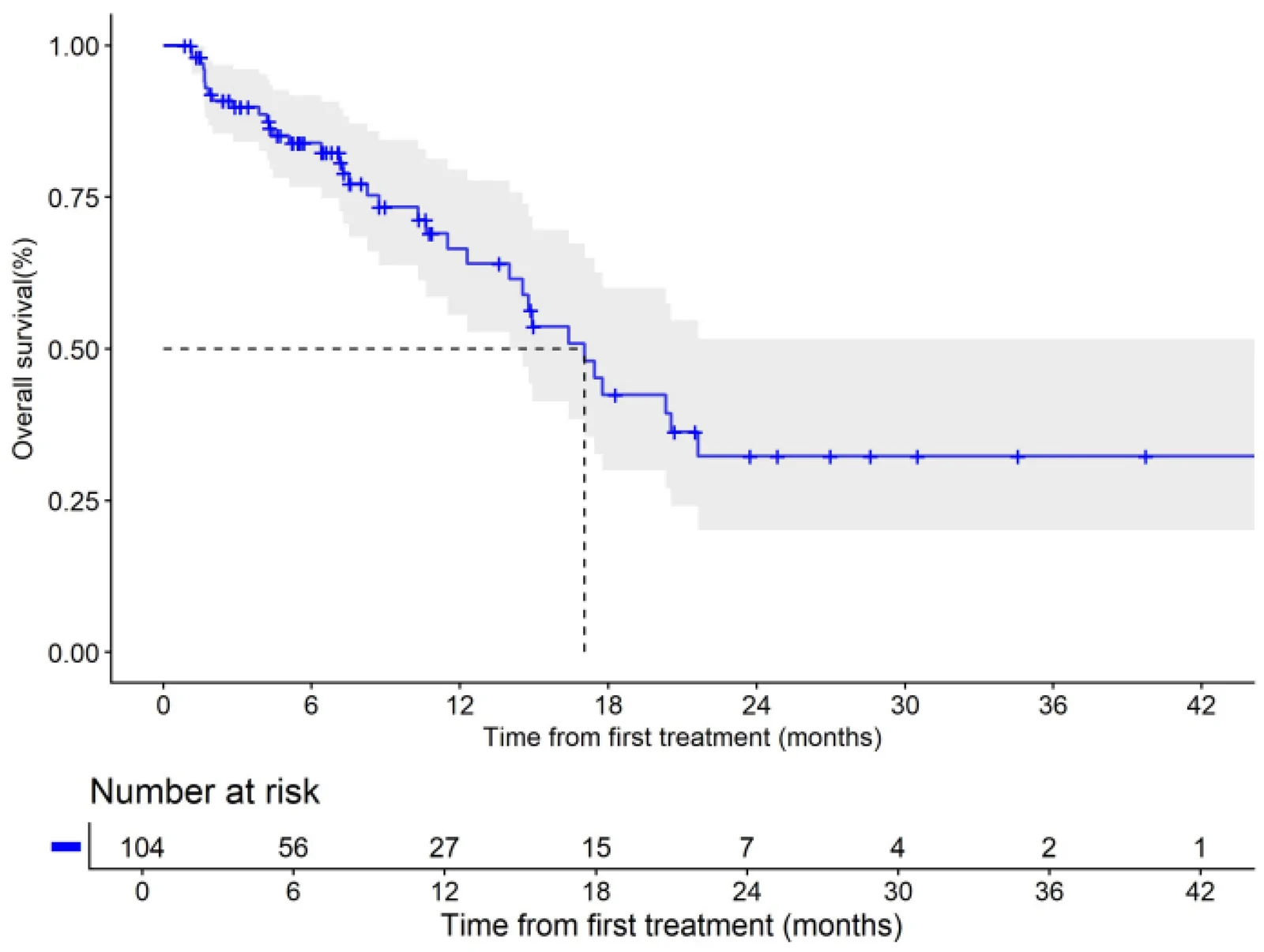
Kaplan–Meier curves for OS in advanced NSCLC patients treated with DEB-BACE. NSCLC, non-small cell lung cancer; DEB-BACE, drug-eluting beads bronchial arterial chemoembolization; OS, overall survival.

**Figure 5:**
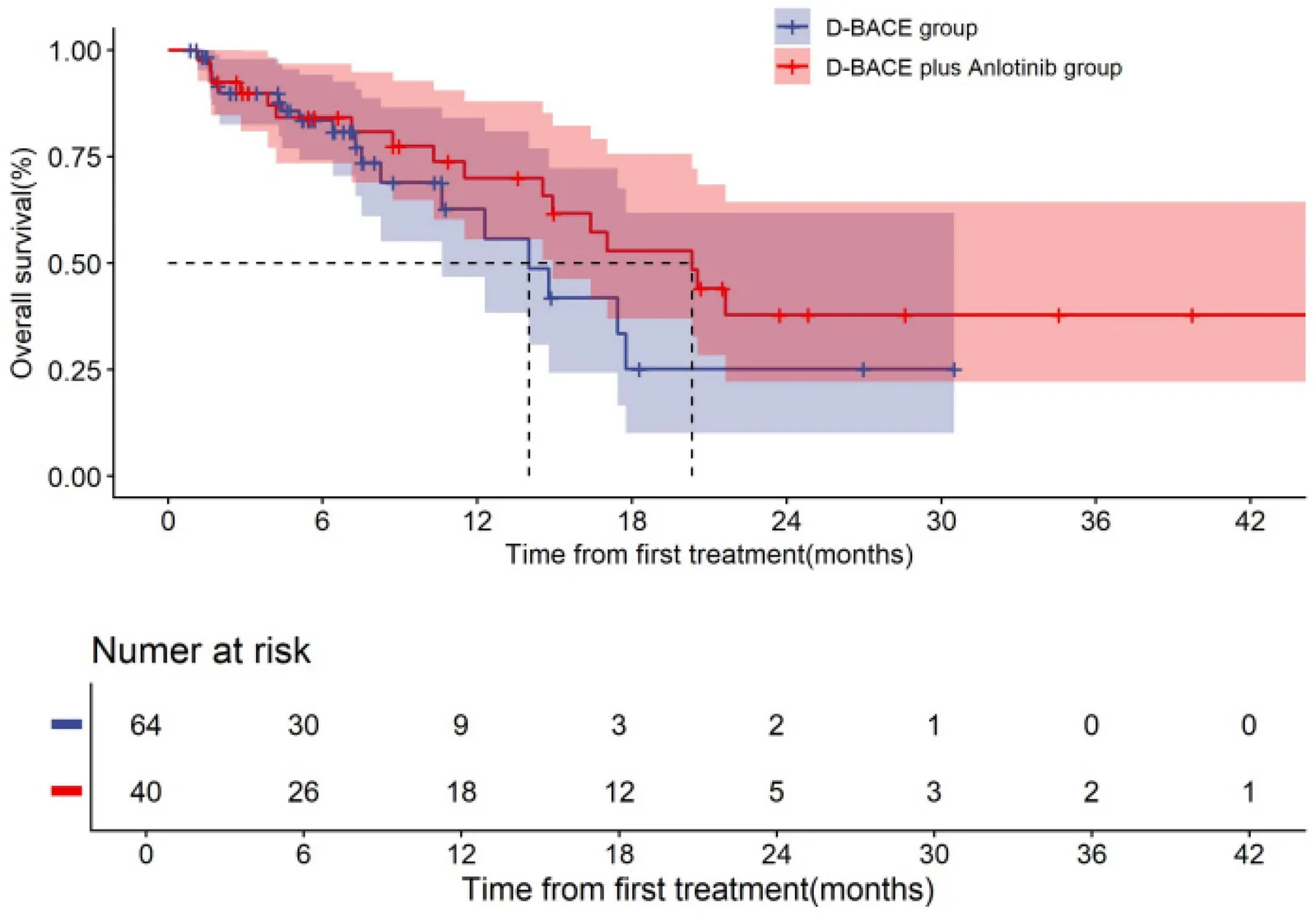
Kaplan–Meier plots of OS in the DEB-BACE monotherapy group and the combination group. The median OS was 20.3 months in the combination group and 14.0 months in the DEB-BACE monotherapy group. DEB-BACE, drug-eluting beads bronchial arterial chemoembolization; OS, overall survival.

For OS, compared with ECOG 0, ECOG 2 exhibited a trend toward poorer survival (*HR* = 2.259, 95% CI: 1.000–5.103, *p* = 0.050), while ECOG 1 was not significantly associated (*HR* = 1.091, 95% CI: 0.429–2.776, *p* = 0.855). Stage IV showed a trend toward worse OS versus stage III (*HR* = 1.893, 95% CI: 0.834–4.299, *p* = 0.127). DEB-BACE combined with anlotinib tended to be associated with more favorable OS compared with DEB-BACE alone (*HR* = 0.500, 95% CI: 0.228–1.094, *p* = 0.083), whereas third- or fourth-line treatment was not significantly associated with OS relative to second-line treatment (*HR* = 0.973, 95% CI: 0.394–2.407, *p* = 0.953), as shown in Table 2, Figure 4 and Figure 5.

### 3.4. Tumor Response Evaluation

According to RECIST 1.1 criteria, tumor responses among 102 patients were assessed. One patient (0.98%) achieved complete response (CR), 12 (11.76%) partial response (PR), 53 (51.96%) stable disease (SD), and 36 (35.29%) progressive disease (PD). The overall response rate (ORR, CR + PR) was 12.75% (13/102) and the disease control rate (DCR, CR + PR + SD) was 64.71% (66/102). Subgroup analysis by treatment arm demonstrated numerically higher ORR (17.95% vs. 9.52%) in the DEB-BACE plus anlotinib group than in the DEB-BACE monotherapy group, although these differences were not statistically significant (ORR: *p* = 0.236; DCR: *p* = 0.244), as shown in Table 3.

### 3.5. Adverse Events

Adverse events (AEs) were classified as DEB-BACE-related or anlotinib-related. No grade 4 AEs or treatment-related deaths were observed. All DEB-BACE-related AEs were grade 1–2, with the most common being cough (15 patients), followed by chest pain (11 patients), nausea (11 patients), and fever (8 patients). For anlotinib-related AEs, grade 1–2 events included hypertension (6 patients), while grade 3 events comprised hemoptysis (3 patients), diarrhea (1 patient), proteinuria (1 patient), and one other grade 3 AE. The overall incidence of grade 3 AEs was 5.8% (6/104). All AEs were manageable with symptomatic treatment or dose modification, and no patients discontinued treatment due to AE intolerance, as shown in Table 4.

## 4. Discussion

Overall, our data suggest that DEB-BACE-based therapy achieved favorable survival outcomes in this high-risk patient population, with a median PFS of 13.9 months and a median OS of 17.0 months for the entire cohort. Although the single-arm design precludes direct comparison with untreated controls, the observed median OS of 17.0 months in our cohort-comprising patients who had progressed after or were ineligible for standard therapies-compares favorably with the historically reported survival of 3–6 months in this heavily pretreated population, suggesting a potential survival signal that warrants further validation. In the subgroup comparison, the DEB-BACE plus anlotinib group showed numerical advantages over the DEB-BACE monotherapy group in ORR (17.95% vs. 9.52%), median PFS (13.9 vs. 10.6 months), and median OS (20.3 vs. 14.0 months). However, DCR was numerically higher in the monotherapy group than in the combination group (69.84% vs. 56.41%), and none of these differences reached statistical significance. ECOG 2 was identified as an independent poor prognostic factor for PFS, while both ECOG 2 and Stage IV disease tended to be associated with worse OS. Importantly, the combination regimen demonstrated an acceptable safety profile, with no grade 4 adverse events or treatment-related deaths; all AEs were manageable and no patient discontinued treatment due to intolerance.

Several relevant studies have reported findings consistent with or distinct from ours, further suggesting the potential efficacy and acceptable safety of DEB-BACE-based regimens in advanced NSCLC. One retrospective study of 36 systemic-therapy-refractory advanced NSCLC patients showed DEB-BACE (*n* = 20) outperformed BAI + PVA embolization (*n* = 16) in OS (12.6 vs. 8.14 months), PFS (4.3 vs. 3.2 months), and ORR (50% vs. 12.5%), with only grade 1 AEs [12]. Another study comparing DEB-BACE and conventional BACE in 41 patients found the DEB-BACE group (*n* = 12) had higher ORR (50.0% vs. 31.0%), DCR (100.0% vs. 72.4%), and longer PFS (6.95 vs. 3.20 months) and OS (28.5 vs. 22.5 months), proving its superiority [10]. A study of 51 patients receiving DEB-BACE then anlotinib had median OS 18.4 months, median PFS 8.4 months, 30-day ORR 21.6%, DCR 100%, mostly mild-to-moderate TRAEs. TNM stage and brain metastasis were risk factors. Its survival outcomes were partially consistent with ours, with differences likely due to treatment sequence and patient baselines [19]. Recent high-level trials further support anlotinib-based therapy: the CAMPASS trial showed that anlotinib plus benmelstobart improved PFS over pembrolizumab in first-line PD-L1-positive NSCLC (*HR* 0.70, *p* = 0.0057) [20], and the FL-ALTER trial showed that adding anlotinib to gefitinib prolonged PFS in EGFR-mutated NSCLC versus gefitinib alone (14.8 vs. 11.2 months; *HR* 0.64, *p* = 0.003), especially in patients with brain metastases [21]. Although these trials enrolled first-line biomarker-selected patients, their consistent PFS benefits strengthen the rationale for combining anlotinib with DEB-BACE.

Advanced NSCLC frequently develops resistance to standard therapies, including chemotherapy, targeted therapy, and immunotherapy. This refractoriness is mainly driven by three critical factors: tumor heterogeneity that promotes drug resistance, vigorous tumor angiogenesis that compensates for treatment-related damage, and systemic micro-metastases that escape local control [7,22,23,24,25,26]. DEB-BACE achieves local tumor control via super-selective infusion of drug-eluting microspheres into tumor-feeding arteries, allowing sustained release of chemotherapeutic agents to induce tumor cell apoptosis. Meanwhile, it permanently embolizes the tumor vasculature, leading to tumor ischemia and necrosis [27,28,29]. However, DEB-BACE cannot eradicate systemic micro-metastases or suppress angiogenesis in residual tumor cells, which may lead to disease progression after treatment [12]. Anlotinib, an oral multi-targeted tyrosine kinase inhibitor (TKI) approved for the third-line treatment of advanced NSCLC, addresses these limitations by targeting key signaling pathways implicated in tumor angiogenesis and proliferation, including vascular endothelial growth factor receptor (VEGFR), platelet-derived growth factor receptor (PDGFR), and fibroblast growth factor receptor (FGFR). This mechanism starves both local and distant tumor cells and prevents disease progression [30,31,32]. Collectively, this combination establishes a “local elimination + systemic control” therapeutic paradigm: DEB-BACE reduces tumor burden and relieves clinical symptoms, whereas anlotinib inhibits angiogenesis-driven disease progression, ultimately yielding synergistic antitumor effects [27,33,34,35,36]. Beyond systemic combination strategies, recent evidence has highlighted the potential of combining transarterial chemoembolization with ablation for primary and secondary lung tumors, further expanding the locoregional treatment armamentarium for advanced lung cancer [37,38].

Safety is a critical consideration in the treatment of advanced NSCLC patients, who often have poor physical status and limited treatment tolerance. In the present study, no grade 4 AEs or treatment-related deaths were reported, and the overall incidence of grade 3 AEs was only 5.8%, which is lower than that reported in previous studies of DEB-BACE or anlotinib monotherapy [19]. All DEB-BACE-related AEs were grade 1–2, with cough, chest pain, nausea, and fever being the most common, consistent with the known safety profile of DEB-BACE [39,40,41]. Anlotinib-related AEs were mainly grade 1–2 hypertension, with only a few grade 3 events (hemoptysis, diarrhea, proteinuria), all of which were manageable with symptomatic treatment or dose adjustment. Importantly, no patients discontinued treatment due to AE intolerance, indicating that both DEB-BACE monotherapy and the combined regimen are well-tolerated in this high-risk patient population.

This study has several limitations that should be acknowledged. First, the retrospective design inherently introduces selection bias, as patients were not randomly assigned to treatment groups, and baseline differences—such as the higher proportion of ECOG 2 and Stage IV disease in the combination group—may have influenced the results. Although we adjusted for ECOG performance status, TNM stage, and prior treatment lines in multivariable Cox regression (Table 2) to minimize confounding, propensity score matching was not performed due to the limited sample size (*n* = 104; combination group *n* = 40), as matching would have further reduced statistical power. We acknowledge that non-randomized allocation and residual confounding are inherent to this retrospective study; therefore, our findings should be interpreted as hypothesis-generating rather than confirmatory. Second, the sample size is relatively small, especially in the combination group, which may have reduced the statistical power to detect significant differences between groups. Third, the follow-up duration is limited, with some survival data censored, which may affect the accuracy of OS and PFS estimates. Fourth, we did not explore potential predictive factors for treatment response, such as genetic mutations or biomarkers, which could help identify patients who might derive greater benefit from the combined regimen. Finally, the available clinical evidence on DEB-BACE-based regimens for advanced NSCLC has been generated predominantly by Chinese research groups. This reflects the current distribution of evidence in this specific field and may limit the generalizability of our findings to other populations; therefore, future international multicenter validation is warranted.

Despite these limitations, this study provides valuable clinical evidence for the use of DEB-BACE-based regimens in advanced NSCLC patients who are refractory to or ineligible for standard treatment. The numerical advantages in survival and tumor response rates with the combined treatment, along with its favorable safety profile, suggest that DEB-BACE combined with anlotinib may be a promising treatment option for this patient population. Future prospective, randomized controlled trials with larger sample sizes, longer follow-up duration, and stratification by baseline prognostic factors (e.g., ECOG performance status, TNM stage) are needed to confirm these findings. Additionally, further studies exploring the underlying mechanisms of the combination treatment, as well as predictive biomarkers, could help optimize treatment selection and improve clinical outcomes for advanced NSCLC patients.

## Figures and Tables

**Table 1:** Baseline characteristics.

Characteristic	Overall *n* = 104	DEB-BACE *n* = 64	DEB-BACE Combined with Anlotinib *n* = 40	*p*-Value
Gender				0.062
Male	81 (77.9)	46 (71.9)	35 (87.5)	
Female	23 (22.1)	18 (28.1)	5 (12.5)	
Age (years)	67.5 (61.0, 74.5)	67.5 (61.0, 74.5)	67.0 (60.5, 74.5)	0.810
Smoking				0.012
Yes	65 (62.5)	34 (53.1)	31 (77.5)	
No	39 (37.5)	30 (46.9)	9 (22.5)	
Alcohol drinking				0.973
Yes	31 (29.8)	19 (29.7)	12 (30.0)	
No	73 (70.2)	45 (70.3)	28 (70.0)	
Body mass index				0.727
<25	76 (73.1)	46 (71.9)	30 (75.0)	
≥25	28 (26.9)	18 (28.1)	10 (25.0)	
Hypertension				0.397
Yes	31 (29.8)	21 (32.8)	10 (25.0)	
No	73 (70.2)	43 (67.2)	30 (75.0)	
Diabetes				0.801
Yes	17 (16.3)	10 (15.6)	7 (17.5)	
No	87 (83.7)	54 (84.4)	33 (82.5)	
Cardio-cerebrovascular diseases				0.801
Yes	17 (16.3)	10 (15.6)	7 (17.5)	
No	87 (83.7)	54 (84.4)	33 (82.5)	
ECOG performance status				0.004
0	29 (27.9)	15 (23.4)	14 (35.0)	
1	47 (45.2)	37 (57.8)	10 (25.0)	
2	28 (26.9)	12 (18.8)	16 (40.0)	
Pathology				0.099
Squamous cell carcinoma	49 (47.1)	25 (39.1)	24 (60.0)	
Adenocarcinoma	51 (49.0)	36 (56.3)	15 (37.5)	
Other	4 (3.8)	3 (4.7)	1 (2.5)	
Preoperative symptoms				0.330
None	61 (58.7)	41 (64.1)	20 (50.0)	
Hemoptysis	31 (29.8)	16 (25.0)	15 (37.5)	
Cough and wheezing	12 (11.5)	7 (10.9)	5 (12.5)	
TNM stage				0.031
stage III	45 (43.3)	33 (51.6)	12 (30.0)	
Stage IV	59 (56.7)	31 (48.4)	28 (70.0)	
Previous treatments				0.029
2-line	25 (24.0)	20 (31.3)	5 (12.5)	
3-line or 4-line treatment	79 (76.0)	44 (68.8)	35 (87.5)	
Number of tumors				0.377
unilateral	85 (81.7)	54 (84.4)	31 (77.5)	
bilateral	19 (18.3)	10 (15.6)	9 (22.5)	
Tumor distribution				0.079
Left lung	57 (54.8)	40 (62.5)	17 (42.5)	
Right lung	30 (28.8)	17 (26.6)	13 (32.5)	
Both lungs	17 (16.3)	7 (10.9)	10 (25.0)	
Maximum diameter of intrapulmonary lesions				0.531
≤5 cm	62 (59.6)	40 (62.5)	22 (55.0)	
>5 cm and ≤7 cm	17 (16.3)	11 (17.2)	6 (15.0)	
>7 cm	25 (24.0)	13 (20.3)	12 (30.0)	
Number of interventions				0.136
1	74 (71.2)	50 (78.1)	24 (60.0)	
2–3	27 (26.0)	13 (20.3)	14 (35.0)	
≥4	3 (2.9)	1 (1.6)	2 (5.0)	
Diameter of microsphere (μm)				0.779
100–300	58 (55.8)	35 (54.7)	23 (57.5)	
300–500	46 (44.2)	29 (45.3)	17 (42.5)	
Extrapulmonary metastasis				1.000
Yes	95 (91.3)	58 (90.6)	37 (92.5)	
No	9 (8.7)	6 (9.4)	3 (7.5)	
Lymph node metastases				0.310
Yes	89 (85.6)	53 (82.8)	36 (90.0)	
No	15 (14.4)	11 (17.2)	4 (10.0)	
Liver metastasis				0.730
Yes	9 (8.7)	5 (7.8)	4 (10.0)	
No	95 (91.3)	59 (92.2)	36 (90.0)	
Brain metastasis				0.154
Yes	5 (4.8)	5 (7.8)	0 (0.0)	
No	99 (95.2)	59 (92.2)	40 (100.0)	
Malignant pleural effusion				0.302
Yes	16 (15.4)	8 (12.5)	8 (20.0)	
No	88 (84.6)	56 (87.5)	32 (80.0)	
White Blood Cell	6.4 (4.8, 7.5)	6.5 (4.8, 7.5)	6.0 (4.7, 7.3)	0.405
Platelet	212.0 (182.0, 260.0)	213.5 (184.0, 250.5)	202.0 (170.5, 265.5)	0.506
Prothrombin Time (PT)	11.3 (10.5, 11.9)	11.1 (10.5, 12.0)	11.4 (10.7, 11.8)	0.468
Hemoglobin	126.0 ± 16.8	125.5 ± 18.6	126.8 ± 13.5	0.685
CYFRA21-1	3.7 (2.4, 9.0)	3.2 (2.4, 7.9)	4.4 (2.4, 10.5)	0.554
NSE	14.7 (12.7, 19.4)	14.4 (12.4, 18.7)	15.4 (13.2, 20.8)	0.321
CEA	3.9 (2.6, 9.7)	3.6 (2.3, 9.0)	4.5 (2.6, 12.3)	0.179

Abbreviations: DEB-BACE, drug-eluting beads bronchial arterial chemoembolization.

**Table 2:** Multivariables Cox regression models for PFS and OS.

Variable	Multivariable PFS			Multivariable OS		
	*HR*	95% CI	*p*	*HR*	95% CI	*p*
Anlotinib plus						
Without	1			1		
With	0.921	0.474~1.789	0.808	0.5	0.228–1.094	0.083
Previous treatments						
2-line	1			1		
3 or 4-line treatment	1.022	0.449~2.327	0.958	0.973	0.394–2.407	0.953
TNM stage						
stage III	1			1		
Stage IV	1.82	0.902~3.671	0.094	1.893	0.834–4.299	0.127
ECOG performance status						
0	1			1		
1	1.404	0.630~3.129	0.407	1.091	0.429–2.776	0.855
2	2.602	1.164~5.817	0.020	2.259	1.000–5.103	0.05

**Table 3:** Tumor response evaluation.

Tumor Response	DEB-BACE, *n* (%), *n* = 63	Combined with Anlotinib, *n* (%), *n* = 39	Overall, *n* (%), *n* = 102	*p*-Value
CR	1 (1.59)	0 (0.00)	1 (0.98)	
PR	5 (7.94)	7 (17.95)	12 (11.76)	
SD	38 (60.32)	15 (38.46)	53 (51.96)	
PD	19 (30.16)	17 (43.59)	36 (35.29)	
ORR	6 (9.52)	7 (17.95)	13 (12.75)	0.236
DCR	44 (69.84)	22 (56.41)	66 (64.71)	0.244

Two patients could not be evaluated for efficacy due to lack of postoperative imaging. Abbreviations: DEB-BACE, drug-eluting beads bronchial arterial chemoembolization.

**Table 4:** Adverse events.

Adverse Events	Grade 1–2	Grade 3	Grade 4
**D-BACE-related AEs**			
Chest Pain	11	0	0
Fever	8	0	0
Cough	15	0	0
Nausea	11	0	0
**Anlotinib-related AEs**	0	1	0
Hypertension	6	0	0
hemoptysis	0	3	0
diarrhea	0	1	0
proteinuria	0	1	0

Data are presented as number of patients (not events).

## Data Availability

The corresponding authors can provide data upon a reasonable request.
